# Indoor Microbiome: Quantification of Exposure and Association with Geographical Location, Meteorological Factors, and Land Use in France

**DOI:** 10.3390/microorganisms8030341

**Published:** 2020-02-28

**Authors:** Steffi Rocchi, Gabriel Reboux, Emeline Scherer, Audrey Laboissière, Cécile Zaros, Adeline Rouzet, Benoit Valot, Sadia Khan, Marie-Noëlle Dufourg, Bénédicte Leynaert, Chantal Raherison, Laurence Millon

**Affiliations:** 1 Department of Parasitology and Mycology, University Hospital, 25030 Besançon CEDEX, France; gabriel.reboux@univ-fcomte.fr (G.R.); escherer@chu-besancon.fr (E.S.); adeline.rouzet@univ-fcomte.fr (A.R.); lmillon@chu-besancon.fr (L.M.); 2 Chrono-Environnement Research Team UMR/CNRS-6249, Bourgogne-Franche-Comté University, 25000 Besançon, France; audrey.laboissiere@univ-fcomte.fr (A.L.); benoit.valot@univ-fcomte.fr (B.V.); 3 INED French Institute for Demographic Studies, ELFE Joint Unit Campus Condorcet 9, 93322 Aubervilliers CEDEX, France; cecile.zaros@ined.fr (C.Z.); marie-noelle.dufourg@ined.fr (M.-N.D.); 4 INSERM Bordeaux Population Health Research Center U1219, Bordeaux University, 33076 Bordeaux, France; sadia.khan@u-bordeaux.fr (S.K.); chantal.raherison@chu-bordeaux.fr (C.R.); 5 Inserm U1168, VIMA Aging and Chronic Disease, 94809 Villejuif, France; benedicte.leynaert@inserm.fr; 6 UMR S 1168, Versailles Saint Quentin University, 78180 Montigny le Bretonneux, France; 7 Department of Pneumology, University Hospital, 33000 Bordeaux, France

**Keywords:** indoor exposure, molds, bacteria, dust mites, qPCR, electrostatic dust collector

## Abstract

The indoor microbial community is a mixture of microorganisms resulting from outdoor ecosystems that seed the built environment. However, the biogeography of the indoor microbial community is still inadequately studied. Dust from more than 3000 dwellings across France was analyzed by qPCR using 17 targets: 10 molds, 3 bacteria groups, and 4 mites. Thus, the first spatial description of the main indoor microbial allergens on the French territory, in relation with biogeographical factors influencing the distribution of microorganisms, was realized in this study. Ten microorganisms out of 17 exhibited increasing abundance profiles across the country: Five microorganisms (*Dermatophagoïdes pteronyssinus*, *Dermatophagoïdes* spp., *Streptomyces* spp., *Cladosporium sphaerospermum*, *Epicoccum nigrum*) from northeast to southwest, two (*Cryptococcus* spp., *Alternaria alternata*) from northwest to southeast, Mycobacteria from east to west, *Aspergillus fumigatus* from south to north, and *Penicillium chrysogenum* from south to northeast. These geographical patterns were partly linked to climate and land cover. Multivariate analysis showed that composition of communities seemed to depend on landscapes, with species related to closed and rather cold and humid landscapes (forests, located in the northeast) and others to more open, hot, and dry landscapes (herbaceous and coastal regions, located in the west). This study highlights the importance of geographical location and outdoor factors that shape communities. In order to study the effect of microorganisms on human health (allergic diseases in particular), it is important to identify biogeographic factors that structure microbial communities on large spatial scales and to quantify the exposure with quantitative tools, such as the multi-qPCR approach.

## 1. Introduction

Individual allergic sensitivity is the main risk factor for asthma development and the early childhood environment is important in the development of allergic diseases [1]. Both protective [2] and harmful effects [3,4,5] of environmental indoor microorganisms have been suggested, but the exact role of bacteria, molds and mites remains unknown, and there is no at-risk or protective threshold available for any species [6]. One explanation is the lack of objective quantification of indoor microbial exposure, which is a major limit in most studies. Moreover, the impact of simultaneous exposure to multiple contaminants (molds, bacteria, and mites) has been poorly studied up to now. 

The consideration of the indoor microbiome is indeed new [7] and the relative implication of factors that structure the composition of microbial communities in the built environment remains unknown [8].

The indoor microbial community is a mixture of microorganisms from various outdoor ecosystems that seed the built environment [9]. Regarding the outside environment, the atmosphere has an incredible richness of microbial diversity, with some microorganisms able to have metabolic activities in clouds [10]. Fungi and bacteria are capable of dispersing over great distances through the effect of wind [11], moving up to thousands of kilometres [12,13]. However, the idea that there can be homogeneity in the distribution of spores in the atmosphere is false. Instead, there are varieties of regional and local atmospheres and, above them, certain atmospheric corridors between remote regions defining a biogeographic distribution of microorganisms [10]. Thus, it is expected that the geographical location of a dwelling (and therefore the associated climatic parameters and type of land use) may influence its microbial contamination [14,15,16,17]. It has been shown, for example, that the geographical position of an outdoor dust sample can be identified, within a median error of 230 km, based on the fungal biome identified [18]. However, geographical location as a factor influencing indoor microbial communities remains largely unstudied, with only three studies that evaluated fungal and/or bacterial contamination across the USA [19,20,21].

In surface ecosystems, biotic and/or abiotic properties of the soil and land use, including type of vegetation cover, modify the diversity of communities to varying degrees [14,22,23,24,25,26]. Meteorological factors, such as temperature, relative humidity, and precipitation, have also been said to influence sporulation and the dispersion of fungi [27]. Snow episodes or low temperatures in sub-Arctic regions have been described as being able to reduce fungal concentrations in outdoor air [28,29]. In addition to these spatial patterns, there is also a temporal and seasonal influence (including season or plant phenological period) on the distribution of microorganisms [17,30]. In temperate climates, peaks of fungal counts are observed in the summer, as in the case of *Cladosporium* spp. [31], or spore concentrations can also differ between days or years, as previously described for *Alternaria* spp. [32]. 

Given the complexity of the indoor exposure, with microorganism biotopes probably defined by a combination of bioclimatic area and ecosystem properties with temporal variation, it is important to analyze parameters that shape this exposure, to better understand the microbiological environment as we should. 

In our first study in 2015, we quantified 10 microorganisms (6 fungal species, 1 family and 2 genera of bacteria, 1 house dust mite) by real-time quantitative PCR (qPCR) in dust samples, collected with electrostatic dust collectors (EDCs) in the dwellings of 3193 children of the ELFE cohort (French nationwide birth cohort “Etude Longitudinale Française depuis l’Enfance”) throughout France [33]. These qPCR targets were chosen for their allergic, infectious, or toxic effect and, at that time, we showed that exposure could be defined by 6 “microorganism cocktail” profiles that had geographical disparities. Thus, in an attempt to more fully analyze the biogeography of indoor microbial life, a spatial analysis was done at a finer resolution than in the first study (21 regions vs. 93 departments). To achieve this goal, statistical methods utilized in ecology to analyze nonlinear data with spatial and/or temporal correlations were used. The qPCR panel was also expanded from 10 to 17 microorganisms (including others mites [34] and yeasts [35] compared to the first study) to increase the potential number of species that may have an effect on children’s respiratory health. First, we described the geographical distribution of selected organisms throughout the country and then we selected those having a significant pattern and focused on outdoor factors (climate and biophysical land use) that could influence this geographical distribution. Thus, by analysing the geographical position of the dwellings, considered to be an essential factor [14,19,36,37,38], we provided the first spatial description of the main indoor microbial allergens on the French territory in relation to outdoor factors influencing the distribution of microorganisms. 

## 2. Material and Methods

### 2.1. Samples and qPCR Method

Sampling was performed using EDC in the dwellings of 3193 children (ELFE subsampling, called EBRA-ELFE (Environnement Biologique et Risque Allergique) at the time of their birth (2011) [33]. EDC were analyzed by qPCR using a panel of 17 targets: 9 molds (*Alternaria alternata, Aspergillus fumigatus, Aspergillus versicolor, Cladosporium sphaerospermum, Penicillium chrysogenum, Stachybotrys chartarum, Trichoderma viride, Chaetomium globosum, Epicoccum nigrum*), 3 bacteria groups (Enterobacteria, Mycobacteria, and *Streptomyces* spp.), 4 dust mites (*Dermatophagoides* spp. (house dust mites, HDM), *D. pteronyssinus* (HDM), *D. farinae* (HDM), *Acarus siro* (storage mites, SM)), and one yeast (*Cryptococcus* spp.). All primers and probes had already been used in our previous studies [33,34,39,40] except the *Cryptococcus* spp. system that was designed for this study. Primers and probes sequences are provided in Table 1. They are available on the Environmental Protection Agency website [41] or were designed by our team or taken from other studies [42,43,44]. 

### 2.2. qPCR Data

To avoid quantification differences between the different targets (due to the fact that qPCRs are designed on repeated regions in the genome), the results were transformed into copy number equivalents. Plasmids containing each targeted sequence were produced using the Zero Blunt^®^ PCR Cloning Kit (Invitrogen, Carlsbad, CA, USA). The copy number was then calculated for each DNA standard dilution as a function of the amount of DNA, Avogadro’s number (6.022.10^23^ molecules/moles), and the length of the plasmid and its insert, according to the formula: number of copies (in ng) = (amount of DNA × 6.022.10^23^)/(length (in bp) × 1.10^9^ × 650). For each DNA standard dilution, DNA concentrations (in fg/µL) were expressed in copy numbers. The coefficients of the lines thus obtained were used as a correction factor to express the qPCR results in copy number.

### 2.3. Climate Data and Land Use

For climate data, we chose those described by Joly et al. who defined the French climates (https://journals.openedition.org/cybergeo/23155). The authors proposed a method for analyzing and processing available climate data (Météo France, the official French meteorological institute) to produce maps that best reflect spatial variations in climate. They described 8 types of climates, defined according to 14 variables (6 temperature and 8 precipitation variables) with monthly data from 1971 to 2000. Thus, we used their constructed variables: annual average temperature, number of days with a temperature below −5 °C, number of days with a temperature above 30 °C, annual temperature range, annual precipitation totals, number of precipitation days in January, number of precipitation days in July, and the ratio between autumn downpour (September + October) and July downpour. 

We also used Corine Land Cover data, a European database of biophysical land use. Fifteen different possible cover types were used: urban fabric; industrial, commercial and transport units; mine, dump and construction sites; artificial, non-agricultural vegetated areas; arable land; permanent crops; pastures; heterogeneous agricultural areas; forests, scrub and/or herbaceous vegetation associations; open spaces with little or no vegetation; inland wetlands; coastal wetlands; inland waters; and marine waters. 

### 2.4. Statistical Analyses

Statistical analyses were carried out in two stages: we observed the distribution of each target independently of the others and examined the entire community through multivariate analyses. All statistical analyses and graphical displays were performed using R [45] and libraries nlme [46], mgcv [47], and mapdata [48].

Spatial distributions of dwelling qPCR targets were described using the spatial coordinates of each French department centroids, according to the 1984 World Geodetic System. These coordinates were denoted by X and Y for longitude and latitude, respectively. A finer spatial grain for dwelling location could not be achieved given the ELFE cohort’s confidentiality rules. 

A first single redundancy analysis (RDA) was performed to assess the structuring effects of geographical position of the dwellings (latitude and longitude coordinates) and month of sampling on microorganism composition. RDA is an extension of principal component analysis (PCA) that models response variables as a function of explanatory variables (in this first RDA: months and X and Y coordinates). Partitioning of variance due to each of the variables could thus be calculated.

Generalized additive mixed models (GAMMs) were used to model the spatial patterns for the different qPCR targets [49]. GAMMs allow nonparametric forms of regression in which smooth functions of the explanatory variables replace the sum of regression coefficients x explanatory variables. Two reasons motivated the use of such models: first, nonlinear relationships between taxa abundances and spatial predictors (geographic coordinates) that were expected could be taken into account using GAMMs through cubic regression splines as a smoothing function. The optimal amount of smoothing was defined by cross-validation and referred to in the model outputs as estimated degrees of freedom (edf), where edf = 1 corresponds to linear relationships and an edf higher than 1 indicates nonlinear relationships. In order to prevent overfitting, maximal edf was set at 4 in the models. Then, the non-independence of samples that were collected at different months could be handled with GAMMs by considering month as a random effect and spatial coordinates as fixed effects in the model. Akaike information criterion (AIC) was used to assess the need to consider month as a random part to improve model performance.

The amount of each target with geographical distribution was modeled with climate and biophysical land use variables. Linear mixed effect models were applied using the nlme function, incorporating a random effect for the month of sampling. The backwards selection method (based on AIC) was used to assess model improvement and to define the final model. Then, the influence of climate and biophysical land use on community composition was assessed through partial redundancy analysis (pRDA), with month of sampling and departments’ centroids (X and Y) considered as covariables to remove their influence. A forward selection was performed to select only significant covariates using the ordiR2step function, with model choice adjusted R^2^ and *p*-value. 

## 3. Results

### 3.1. qPCR Analysis

The analysis of the contamination of the 3143 dwellings was carried out after excluding housing from the Corsica department and that for which parents did not complete the questionnaires. *C. globosum* was not included in the analyses because there were more than 95% of EDCs with a negative result for this target. The number of positive EDCs and the median and maximum copy number for each target are presented in Table 2. 

### 3.2. Spatio-Temporal Distribution of Microorganisms

**Figure 1:**
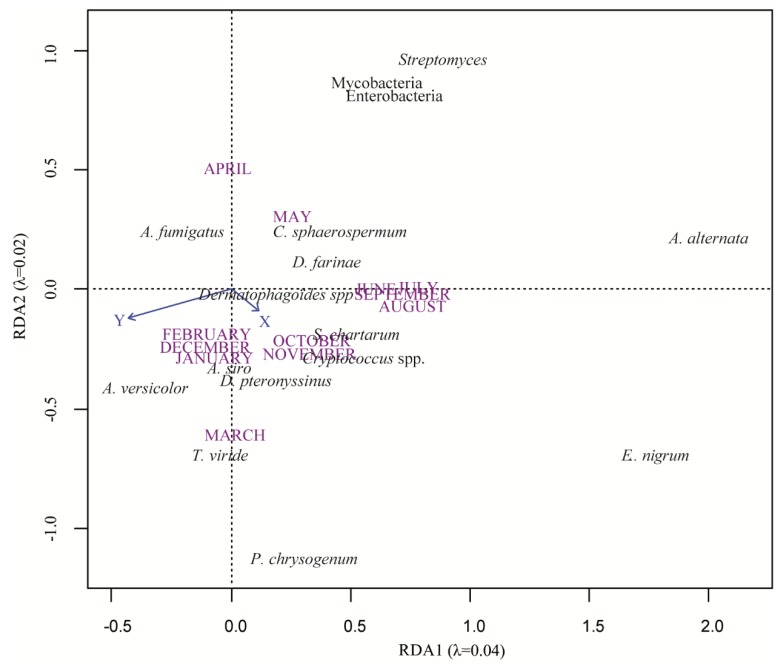
Redundancy analysis (RDA) plot showing target microorganisms and spatio-temporal (X and Y coordinates and months of samplings) distribution. Microorganisms are in black, coordinate variables in blue, and months in purple. Solid arrows indicate quantitative variables (X: longitude and Y: latitude). Months of samplings are qualitative variables and are represented without arrows.

According to the first RDA (structuring effects of geographical position (X: longitude and Y: latitude) and month of sampling), the microorganism distribution was structured by X coordinates (*p* < 0.001), Y coordinates (*p* < 0.001), and months of sampling (*p* < 0.001) (Figure 1). These three variables explained 9% of microorganism variability and partitioning of variance showed that X and Y coordinates each accounted for 1% of variability and month of sampling for 7%.

**Figure 2:**
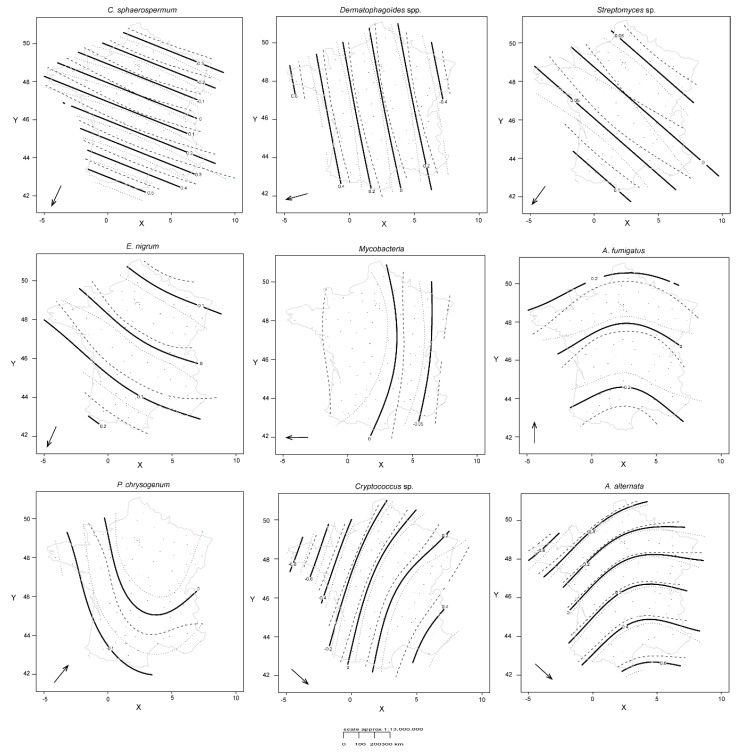
Spatial patterns for log-transformed copy number of qPCR targets for nine microorganisms exhibiting significant spatial variability. As *Dermatophagoïdes* spp. and *D. pteronyssinus* targets showed the same distributions, only one of the two is represented. Solid lines represent smoother contours (smoothing component f(Xi,Yi), with X: longitude and Y: latitude) and the dotted lines are 95% confidence bands. Arrows indicate the direction of the abundance gradient from lower to higher abundances.

In GAMMs, the consideration of sampling month as a random effect significantly improved model predictions (reduction in the AIC value). Among the 17 qPCR targets, 10 exhibited significant spatial variability, although the amount of variability explained by the spatial structure remained rather restricted (Table 3). Non-linear patterns were found for some targets (Figure 2), especially for *A. alternata* (with greater effective degrees of freedom, edf, in Table 3). The higher the edf, the more non-linear the smoothing spline is. 

Although some microorganisms showed distribution gradients in the same geographical direction, the correlation analysis (Pearson coefficient) was not significant. Five microorganisms had northeast to southwest increasing abundance profiles (on a line from Reims to Bordeaux): *D. pteronyssinus* and whole *Dermatophagoïdes* spp. (260° SW), *Streptomyces* spp. (220° SW), *C. sphaerospermum* (200° SW), and *E. nigrum* (200° SW). The mean of the azimuth of this group was 230° SW. Mycobacteria had an east to west increasing profile (270° W), *A. fumigatus* showed an increasing gradient from south to north (0° N), and a south to northeast gradient was found for *P. chrysogenum* (40° NE). *Cryptococcus* spp. and *A. alternata* exhibited an increasing gradient from northwest to southeast (130° SE) (a line from Brest to Nice). The seven other microorganisms (*S. chartarum*, *A. versicolor*, *T. viride*, *D. farinea*, *A. siro*, Enterobacteriaceae) appeared to be evenly distributed throughout the country. 

For microorganisms that have the same gradient direction (*C. sphaerospermum* and *E. nigrum*), spatial patterns of microorganisms showed nuances that were more or less fine (smoother contours and confidence intervals quite narrow in Figure 2).

### 3.3. Climate and Biophysical Land Use for Each Individual Target

Twenty-two variables (8/8 for climate and 14/15 for land use) were considered linked to the qPCR targets that showed spatial variability in the previous section. Table 4 presents the results of the backward selection and the direction of the relationship (positive or negative) for each individual target. The coastal wetland factor was not selected in any model. 

Some qPCR targets were associated with temperature parameters: some were linked to the number of warm days, such as *C. sphaerospermum*, with a positive relation to the number of days >30 °C and a negative relation to the number of days < 5 °C. For others, such as *A. alternata,* distribution was linked to the average annual temperature and annual temperature range (difference between average temperature in July and January). In contrast, *Dermatophagoïdes* spp. were also linked to average annual temperature, but not to annual temperature range. Concerning precipitation, the number of rainfalls in January was the variable that was most frequently related to the distribution of microorganisms, with 5 positive (*C. sphaerospermum*, *Streptomyces* spp., Mycobacteria, *A. fumigatus*, *P. chrysogenum*) and 2 negative (*Cryptococcus* spp., *A. alternata*) relationships.

For biophysical land use, the prairie variable was the most frequently related to the distribution of microorganisms, with two positive (*C. sphaerospermum*, *A. fumigatus*) and seven negative (*Dermatophagoïdes* spp., *Streptomyces* spp., *E. nigrum*, Mycobacteria, *P. chrysogenum*, *Cryptococcus* spp., *A. alternata*) relationships. In contrast, mines, landfills, and construction sites were only positively related to *A. fumigatus.*

### 3.4. Microorganism Community

**Figure 3:**
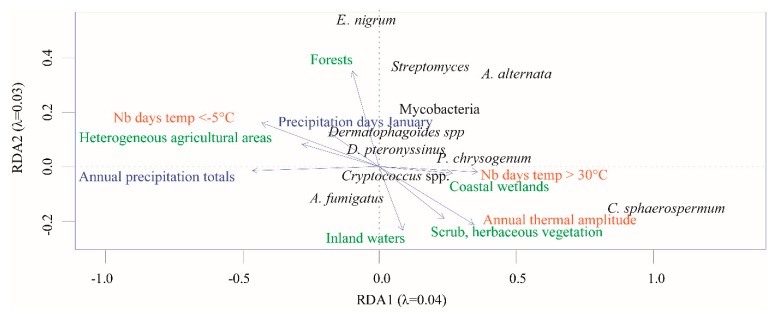
Partial redundancy analysis (pRDA) triplot showing target microorganisms and model selected climate and landscape variables. Microorganisms are in black, temperature variables in orange, precipitations variables in blue, and land cover in green.

Partial redundancy analysis (pRDA) triplot, depicting the structuring effect of climate and biophysical land use on microorganism communities, is presented in Figure 3. Considered together, the month of samplings and departmental coordinates (X and Y) accounted for 16% of the dataset variance.

Among the 23 variables of climate and biophysical land use, 9 were selected by a forward selection model and were significant (*p* < 0.05), thus explaining a part of the microorganism distribution in dwellings. However, these variables could only account for a small fraction of the variance (1.6%). In Figure 3, explanatory variables constrain the first ordination axes in order to interpret the species matrix. Thus, from the observations that emerged from this analysis, *E. nigrum* was mainly associated with areas covered by forests, whereas *C. sphaerospermum* was linked to annual temperature range and scrub/herbaceous vegetation areas. *Dermatophagoïdes* spp. were related to the number of days of precipitation in January. These links were not the same in the linear mixed effects models when microorganisms were analyzed individually and not included in a community. More generally, it was as if communities were constrained with a gradient ranging from closed and rather cold and humid landscapes (forests) (on the left side of the graph in Figure 3) to more open, hot, and dry landscapes (herbaceous and coastal). 

## 4. Discussion

The present study addressed the biogeography of indoor microbial exposure, providing an assessment of the microbial composition of French dwellings in terms of molds, bacteria, and dust mites using a single standardized quantification method (qPCR). Given the importance of seasonality on the distribution of microorganisms, the purpose of this paper was to be able to assess the respective weight of geographical location by taking into account the month effect by means of statistical processing (GAMMs and pRDA) and then to evaluate the influence of, probably, the two most important parameters (climate and land cover). Analysis showed that the geographical location of dwellings induces variations in the quantification of different microbial communities (10 out of 17 microorganisms), which is partly linked to patterns of biogeography and climate. 

While linear regression is an important tool for statistical analysis, it may be interesting to look at other methods that may be more adapted to biology/ecology, which are areas that do not necessarily have to follow linear relationships. To assess the distribution of each microorganism individually, we used GAMMs, which allowed us to model nested data and spatio-temporal correlation structures in count data and is a popular approach to modeling complex structures in ecological data or pollution studies [49].

We then performed a redundancy analysis, commonly used to simultaneously analyze the effects of multiple environmental factors on multiple species. This made it possible to represent the most important and interpretable environmental gradients available and, more importantly, to provide an overall descriptive analysis, rather than multiple univariate analyses. In our pRDA, month of sampling and X and Y coordinates were considered as condition variables so that their influence was removed prior to assessing the effect of climate and land cover variables. This procedure examined the determinants of community variability once the temporal (month of sampling) and spatial (X and Y) variability were accounted for.

With the statistical analyses conducted, we demonstrated that, if we consider the individual microorganisms (linear models) or community of microorganisms (pRDA), the climatic and occupancy variables that are selected in the models and are linked to the distribution of the microorganism(s) are different. 

Outdoor geographical clusters of microbial communities have already been documented on local, regional, and continental scales [14] and geography is also a major factor that influences indoor fungal communities [36]. Thus, we expected that some qPCR targets would be dependent on the geographic coordinates of the dwellings, such as *Alternaria*, *Cladosporium*, *Penicillium*, and *Aspergillus*, which are among the most common genera found indoors and were previously described as strongly influenced by meteorology. A recent publication, conducted on a European scale reported that outdoor concentrations of *Alternaria* and *Cladosporium* spores differed between sites and years of sampling [27]. With our work, we have eliminated the month of sampling effect by taking it into account in the models and have shown that, at the scale of France, we also observe differences in concentrations of *Alternaria* and *Cladosporium* according to geographic locations and this is the case within dwellings as well. In contrast, geographical gradients were less predictable for other targets, but this is probably due to our “mistaken” vision of reality that is linked to cultivation methods. For example, the *Epicoccum* genus is rarely found in studies that use culture as a means of studying fungal exposure, particularly due to difficulties with culture and sporulation on classical media. However, this genus is increasingly reported in studies, including targeted metabarcoding approaches, that place it in first place among fungi [35,40,50,51,52]. Another advantage of using biomolecular methods, and particularly qPCR, is the ability to quantify different types of microorganisms with the same technique without competition between microorganisms (as in the case of culture investigations). Moreover, qPCR takes into account live and dead cells, which represent a significant part of fungal exposure, also influencing immunological responses [53,54]. We were thus able to show that there were different concentration gradients in a given geographical area for yeasts (*Cryptococcus* spp.), bacteria (Mycobacteria and *Streptomyces* spp.), and HDM (*Dermatophagoïdes* spp. and *D. pteronyssinus*). 

Some climate parameters, land use, and/or vegetation cover have been linked to the geographical pattern of microorganisms in univariate analysis, but, above all, the pRDA allows us to place some targeted species on a scale of landscapes and climates (closed and rather cold and humid landscapes to more open, hot and dry landscapes). We have been working on land use and vegetation cover data collected for 2011. For climate, we selected 14 climate variables (6 temperature and 8 precipitation variables), defining 8 French climates. 

Some microorganisms not highlighted by our statistical analysis (*S. chartarum*, *A. versicolor*, *T. viride*, *D. farinea*, *A. siro*, Enterobacteriaceae) are probably not or are less driven by the outdoor parameters we studied. They can be driven by other outdoor factors (wind or vapor pressure [27]) or maybe by indoor characteristics, such as thermo-isolation and ventilation, infiltration, and water damage, but also occupants (pets, humans, plants), the type of use of the indoor location, architecture, and materials [1,55,56,57]. For example, it would not be surprising that some microorganisms, such as *S. chartarum*, described as linked to water damage [58] or *A. siro*, found in the foodstuffs in our kitchens [59], are not related to the landscape cover type. 

Given the information mentioned here, the complexity of exposure and the influence of the various factors, which are probably interconnected, it seems difficult to reason in terms of exposure to a single microorganism when we want to address the issue of allergic diseases. Above all, nothing is single-factorial. On the other hand, as long as we use qualitative methods to measure the presence of microorganisms, we can certainly believe that “everything is everywhere”. However, quantification methods show us that we are dealing with various concentrations in different environments. Thus, to assess an allergic risk related to microbial exposure, it would undoubtedly be useful to build “composite” indices, mixing exposure measurements, environmental factors (geography, climate, vegetation), and housing factors (humidity, temperature, ventilation).

## Figures and Tables

**Table 1:** Primers and probes sequences (and their references) used in this study.

Targets and Designers [Reference]		(5’–3’) Sequences	
Molds	*Alternaria alternata* designed by EPA [41]	Forward primer	GGCGGGCTGGAACCTC
		Reverse primer	GCAATTACAAAAGGTTTATGTTTGTCGTA
		Probe	TTACAGCCTTGCTGAATTATTCACCCTTGTCTTT
	*Aspergillus fumigatus* designed by EPA [41]	Forward primer	GCCCGCCGTTTCGAC
		Reverse primer	CCGTTGTTGAAAGTTTTAACTGATTAC
		Probe	CCCGCCGAAGACCCCAACATG
	*Aspergillus versicolor* designed by EPA [41]	Forward primer	CGGCGGGGAGCCCT
		Reverse primer	CCATTGTTGAAAGTTTTGAcTGATCTTA
		Probe	AGACTGCATCACTCTCAGGCATGAAGTTCAG
	*Cladosporium sphaerospermum* designed by EPA [41]	Forward primer	ACCGGCTGGGTCTTTCG
		Reverse primer	GGGGTTGTTTTACGGCGTG
		Probe	CCCGCGGCACCCTTTAGCGA
	*Epicoccum nigrum* designed by EPA [41] #	Forward primer	TTGTAGACTTCGGTCTGCTACCTCTT
		Reverse primer	TGCAACTGCAAAGGGTTTGAAT
		Probe	CATGTCTTTTGAGTACCTTCGTTTCCTCGGC
	*Penicillium chrysogenum* Modified from EPA [40]	Forward primer	TGCCTGTCCGAGCGTCATT
		Reverse primer	CCCCCGGGATCGGAG
		Probe	CCAACACACAAGCCGTGCTTGAGG
	*Stachybotrys chartarum* designed by EPA [41]	Forward primer	TCCCAAACCCTTATGTGAACC
		Reverse primer	GTTTGCCACTCAGAGAATACTGAAA
		Probe	CTGCGCCCGGATCCAGGC
	*Trichoderma viride* designed by EPA [41] #	Forward primer	CCCAAACCCAATGTGAACCA
		Reverse primer	TCCGCGAGGGGACTACAG
		Probe	CCAAACTGTTGCCTCGGCGGG
	*Chaetomium globosum* designed by EPA [41] #	Forward primer	CCGCAGGCCCTGAAAAG
		Reverse primer	CGCGGCGCGACCA
		Probe	AGATGTATGCTACTACGCTCGGTGCGACAG
Yeasts	*Cryptococcus* spp. designed for this study #	Forward primer	CCTGCGGAAGGATCATTAATG
		Reverse primer	GCACAGGTGTTATGGATATGATGTG
		Probe	TTGACCGTCTGTCGAGCTTGCTCACA
Mites	*Acarus siro* Designed by our team [34] #	Forward primer	CGCAAACTGTGGTGCGAGTA
		Reverse primer	GCTCCTTGGTCCGTGTTTCA
		Probe	TCGGTCTCCACCCGACCCGTC
	*Dermatophagoïdes* spp. Designed by our team [34]	Forward primer	TGTTGTGGTTAAAAAGCTCGTAGTTG
		Reverse primer	ATGCGATAATCTGCTCAGTATGACA
		Probe	CAGCTCATGTATGGCGGTCCACCTG
	*Dermatophagoïdes farinea* Designed by our team [34] #	Forward primer	CACACATTCAACCAGAGTGGTACTT
		Reverse primer	GGCTAACACTCCCCCTAGTTTAGA
		Probe	CGCTTACGCGATCCTACGAGCCATT
	*Dermatophagoïdes pteronyssinus* Designed by our team [34] #	Forward primer	CATCCAACCAGAGTGGTATTTCC
		Reverse primer	GCTATTGCGCATACTCCACCTA
		Probe	TATGCAATCCTTCGGGCTATCCCATCA
Bacteria	Enterobacteriaceae designed by Sen and Asher [42]	Forward primer	GGCGGCAGGCCTAAC
		Reverse primer	CAGGCAGTTTCCCAGACATTACT
		Probe	AGCAAGCTCTCTGTGCTACCGCTCGA
	Mycobacteria designed by Torvinen et al. [43]	Forward primer	GATGCAACGCGAAGAACCTT
		Reverse primer	TGCACCACCTGCACACAGG
		Probe	CCTGGGTTTGACATGCACAGGACG
	*Streptomyces spp.* designed by Rintala and Nevalainen [44]	Forward primer	GCCGATTGTGGTGAAGTGGA
		Reverse primer	GTACGGGCCGCCATGAAA
		Probe	ATCCTATGCTGTCGAGAAAAGCCTCTAGCG

EPA: Environmental Protection Agency. The seven additional qPCR targets to the 10 used in the first study published in 2015 [33] are notified by “#”.

**Table 2:** Positivity and quantification of each target by qPCR in the 3143 electrostatic dust collectors (EDC) analyzed.

qPCR Targets		Number of Positive EDCs	Median Value (copy/µL)	Max Value (copy/µL)
Fungi (molds and yeasts)	*E. nigrum*	2763	76	39106
	*A. alternata*	2752	66	32634
	*P. chrysogenum*	2660	9	19169
	*C. sphaerospermum*	2195	8	12162
	*A. versicolor*	2307	2	5671
	*A. fumigatus*	1468	0	5138
	*T. viride*	1390	0	1536
	*S. chartarum*	848	0	333
	*Cryptococcus* spp.	2314	<1	7
Bacteria	Enterobacteriaceae	3086	134	78163
	Mycobacteria	3053	235	12511
	*Streptomyces*	2879	51	5887
Dust mites	*Dermatophagoïdes* spp.	1505	<1	97673
	*D. pteronyssinus*	1908	0	9657
	*D. farinae*	2192	<1	1330
	*A. siro*	2300	<1	348

**Table 3:** Detailed output on the smoothers and parametric terms in the Generalized additive mixed models (GAMMs).

qPCR Targets		*p*	edf	F	adjR^2^
Molds	*A. alternata*	<0.001 *	2.954	162.6	0.12
	*A. fumigatus*	<0.001 *	2.7	10.54	0.00909
	*E. nigrum*	<0.001 *	2.345	10.85	0.0069
	*C. sphaerospermum*	<0.001 *	2	59.59	0.0418
	*P. chrysogenum*	0.028 *	2.738	4.472	0.00212
	*S. chartarum*	0.122	2.853	1.808	0.00119
	*A. versicolor*	0.126	2	2.071	0.000853
	*T. viride*	0.794	2	0.231	−0.000452
Yeasts	*Cryptococcus* spp.	<0.001 *	2.852	42.88	0.0391
Dust mites	*Dermatophagoïdes* spp.	<0.001 *	2.122	39.51	0.0286
	*D. pteronyssinus*	<0.001 *	2	15.77	0.00918
	*D. farinea*	0.672	2.248	0.44	−0.000307
	*A. siro*	0.779	2	0.249	−0.000525
Bacteria	Enterobacteriaceae	0.12	2.25	1.823	0.00139
	Mycobacteria	0.009 *	2.694	3.544	0.00456
	*Streptomyces*	<0.001 *	2	11.71	0.00851

*p* (*p*-value) is given for each GAMM with “*” when <0.05; edf: estimated degrees of freedom (can be viewed as an estimation of the strength of the smoothness considered in the models); F: F-statistics; adjR^2^: Adjusted R-squared.

**Table 4:** Climate and biophysical land use contributing to the distribution of microorganisms (linear mixed-effects models).

	*C. sphaerospermum*	*Dermatophagoïdes* spp.	*Streptomyces*	*E. nigrum*	Mycobacteria	*A. fumigatus*	*P. chrysogenum*	*Cryptococcus* spp.	*A. alternata*
Annual average temperature		+	−	+		−			+
Number of days temperature < −5 °C	−		−			−	+	+	
Number of days temperature >30 °C	+		+		+				
Annual temperature range *		−			−	+			+
Total annual precipitation		+	−		−				
Number of precipitation days in January	+		+		+	+	+	−	−
Number of precipitation days in July							−		
Ratio between autumn ** and July precipitations		−	−	−	−				−
Urbanized areas		−	−	−	−	+		−	−
Industrial or commercial areas	+	−		−					
Mines, landfills, and construction sites						+			
Artificial, non-agricultural green spaces	+			−					
Arable land	+	−	−	−	−			−	−
Permanent crops	+	−	−	−	−			−	−
Prairies	+	−	−	−	−	+	−	−	−
Heterogeneous agricultural areas		−		−	−	+	−	−	−
Forests	+	−		−	−	+		−	−
Scrub and/or herbaceous vegetation associations	+	−		−		+			
Open spaces, with little or no vegetation		−		−		+		−	−
Inland wetlands	+			−					
Inland waters	+	−	−	−	−		−		
Maritime waters				−					

Positive relations are indicated by “+” and negative by “−”. Empty cells mean that the variable was removed from the model (ordiR2step function). * Annual temperature range measures the difference between the average temperature in July and January. **Autumn precipitations: September + October.
